# Pneumonia at a Base Hospital

**Published:** 1918-11

**Authors:** 


***Pneumonia at a Base Hospital.*** By A. A. SMALL (Chicago), Camp Pike, Little Rock, Ark. *Journal of the American Medical Association*, Aug. 31, 1918.

The author gives an account of the pneumonia morbidity and fatality at Camp Pike, Ark., between September, 1917, and April 27, 1918. In this period there were 1,285 pneumonia patients; 857 of these had lobar pneumonia, and 428 had bronchopneumonia. In the beginning the cases were mild, and in both forms corresponded to the ordinary types seen in civil life. The mortality was low. As winter came on, however, the proportion of bronchopneumonia increased, and the mortality also increased, especially that of bronchopneumonia, which showed 53 per cent. fatal in January. After that, the death percentages declined, being nearly parallel at the close of the period mentioned. It is difficult, Small says, to give a definite reason for the difference in mortality in midwinter and the later months compared with that of the first two months. The antecedent measles may have been a factor in the bronchopneumonias, and also in a number of the streptococcus cases. The author thinks that the virulence of the organism must undoubtedly be increased by its passage through the human host. Many of the cases of bronchopneumonia began with the most trivial subjective symptoms, and practically no objective signs. A few fine moist râles, usually at the back and near the angle of the scapula, were often the only physical signs present, but in another twelve or twenty-four hours there would be marked consolidation in the same area, increase of the moist râles, and often bronchovesicular or tubular breathing. Fever is usually not high, and the pulse not rapid. Peculiar as it may seem, the majority of these patients showed no respiratory distress or dyspnea until near death. Cyanosis is rare, and expectoration is late and moderate. In contrast to the majority, we have an acute fulminating type, which is illustrated by the case history of a patient who entered the hospital at 7 p. m. one day, and died the next morning at 6 a. m. He had been drilling the day before and complained of only slight ailments. Empyema occurred in slightly over 9 per cent. of the total number of pneumonia patients. The types of empyema have been described by Dr. McKenna. Sputum examinations in 48 per cent. of the pneumonias showed the pneumococcus, Type I in 21 per cent., Type II in 34 per cent., and 45 per cent. of Type IV. A number of patients were received with the diagnosis of meningitis, but the spinal fluid examination corrected this. The three most patent causative factors, Small believes, are the large number of men suffering from measles, the large amount of dust, and the carelessness of the men in their barracks causing minor respiratory disorders.

